# Bayesian Network Analysis of Lymphatic Filariasis Serology from Myanmar Shows Benefit of Adding Antibody Testing to Post-MDA Surveillance

**DOI:** 10.3390/tropicalmed7070113

**Published:** 2022-06-21

**Authors:** Benjamin F. R. Dickson, Jesse J. R. Masson, Helen J. Mayfield, Khin Saw Aye, Kyi May Htwe, Maureen Roineau, Athena Andreosso, Stephanie Ryan, Luke Becker, Janet Douglass, Patricia M. Graves

**Affiliations:** 1 College of Medicine and Dentistry, James Cook University, Cairns, QLD 4870, Australia; 2 College of Public Health, Medical and Veterinary Sciences, James Cook University, Cairns, QLD 4870, Australia; j.masson@uq.net.au (J.J.R.M.); maureenroineau@wanadoo.fr (M.R.); athena.andreosso@qimrberghofer.edu.au (A.A.); stephanie.ryan1@jcu.edu.au (S.R.); luke.becker@jcu.edu.au (L.B.); jan.douglass@jcu.edu.au (J.D.); patricia.graves@jcu.edu.au (P.M.G.); 3 School of Public Health, Faculty of Medicine, The University of Queensland, Brisbane, QLD 4072, Australia; h.mayfield@uq.edu.au; 4 Department of Medical Research, Ministry of Health and Sports, Yangon 11191, Myanmar; ksadmr@gmail.com (K.S.A.); kyimaywin31@gmail.com (K.M.H.)

**Keywords:** lymphatic filariasis, LF, Myanmar, elimination, antigen, antibody, serology, Bayesian networks

## Abstract

The elimination of lymphatic filariasis (LF) is achieved through repeated mass drug administration (MDA) of anti-filarial medications, which interrupts transmission and prevents new infections. Accurate transmission assessments are critical to deciding when to stop MDA. Current methods for evaluating transmission may be insufficiently sensitive, resulting in post-MDA resurgence. We, therefore, evaluated potential diagnostic testing scenarios for post-MDA surveillance. Data were used from two surveys (a household cluster and a cohort) conducted in an area of Mandalay Region, Myanmar, with ongoing transmission following several rounds of MDA. First, age- and sex-adjusted seroprevalence were estimated for the area using the household survey. Next, three Bayesian networks were built from the combined datasets to compare antigens by immunochromatic testing (ICT) and/or Og4C3 enzyme-linked immunosorbent assay (ELISA) and antibody (Ab) detection methods (Wb123 or Bm14 Ab ELISA). The networks were checked for validity and then used to compare diagnostic testing scenarios. The adjusted prevalence from the household survey for antigen, Wb123 Ab and Bm14 Ab were 4.4% (95% CI 2.6–7.3%), 8.7% (5.96–12.5%) and 20.8% (16.0–26.6%), respectively. For the three networks, the True Skill Statistic and Area Under the Receiver Operating Characteristic Curve for antigen, Wb123 and Bm14 Ab were 0.79, 0.68 and 0.55; and 0.97, 0.92 and 0.80, respectively. In the Bayesian network analysis, a positive case was defined as testing positive to one or more infection markers. A missed result was therefore the probability of a positive case having a negative test result to an alternate marker. The probability of a positive case prior to any testing scenario was 17.4%, 16.8% and 26.6% for antigen, Wb123 Ab and Bm14 Ab, respectively. In the antigen-only testing scenario, the probability of a missed positive LF result was 5.2% for Wb123 and 15.6% for Bm14 Ab. The combination of antigen plus Bm14 Ab testing reduced the probability of missing a positive LF case as measured by Wb123 Ab to 0.88%. The combination of antigen plus Wb123 Ab was less successful and yielded an 11.5% probability of a missed positive result by Bm14 Ab testing. Across scenarios, there was a greater discordance between Bm14 and both antigen and Wb123 Ab in the 1–10 age group compared to older ages. These findings suggest that the addition of Bm14 Ab improves the sensitivity of LF testing for current or past infection. The combination of antigen plus Bm14 Ab should therefore be considered for inclusion in post-MDA surveillance to improve the sensitivity of transmission surveys and prevent the premature cessation of MDA.

## 1. Introduction

Lymphatic filariasis (LF) is a vector-borne helminth infection that is transmitted by a variety of mosquito genera. Adult worms live in the human lymphatic system and produce microfilariae (Mf) which circulate in peripheral blood. Infection with LF damages the lymphatic system resulting in acute adenolymphangitis attacks and chronic swelling of limbs and genitals, which can become irreversible and cause severe morbidity and disability. In 2020, 51.4 million people in 72 endemic countries were estimated to be infected [1].

The Global Programme to Eliminate LF has been delivering annual mass drug administration (MDA) of anti-filarial medication for over 20 years. Population surveys for LF infection are crucial to the programme and are used at three time points: (1) to determine whether areas are endemic and need MDA, (2) to decide when MDA should stop, and (3) to determine whether elimination (interruption of transmission) can be validated. To date, 17 countries have been certified by the World Health Organization (WHO) to have eliminated LF as a public health problem. This requires passing a series of surveillance activities, including Transmission Assessment Surveys (TAS) on 6–7-year-old children [2,3].

Blood tests for surveillance both pre- and post-cessation of MDA and after validation of elimination are usually conducted using tests for circulating filarial antigen. Rapid tests available include the immunochromatographic test (ICT), now superseded by the filariasis test strip (FTS) [4]. An alternative is an enzyme-linked immunosorbent assay (ELISA) such as the Og4C3 test [5]. These tests all detect antigen that is produced by adult worms in the lymphatic system. The rapid tests are done on whole blood, whereas the ELISA antigen tests can be done using serum, plasma or dried blood spots [6]. Since adult worms need to be mature and mated before producing Mf, antigen detection tests are roughly 2–4 times more sensitive than examining blood slides for Mf, although the ratio between the two measures depends on prevalence and recent history of MDA [7].

It is known that antibodies to LF, including Bm14 and Wb123, appear very early in those infected by LF [8], are present at higher prevalence when compared to antigens [9], and persist for many months and even years after treatment [10]. Thus, the presence of antibodies against filarial antigens, especially in young children, may be a more sensitive method of detecting remaining transmission as elimination approaches, thus avoiding premature cessation of MDA.

South-East Asia is highly endemic for LF [11,12], but significant progress in reducing prevalence has been made in recent decades [13]. Three countries in the WHO South-East region (Thailand, Sri Lanka and Maldives) have reached validation of elimination [1].

Myanmar has one of the highest burdens of filariasis in South-East Asia, with 41 million (80% of the population) at risk [11,14]. It is distributed predominantly in the central and western dry zones, where baseline (pre-MDA) prevalence was 20–30% [11,15,16,17]. *Wuchereria bancrofti* is the sole cause of filariasis in the country, where it is transmitted by the mosquito *Culex quinquefasciatus* [11,18].

Myanmar commenced an MDA program with diethylcarbamazine and albendazole in 2001 [14,15]. Since then, coverage has been progressively expanded, reaching all endemic districts by 2015 [17]. The program has faced several challenges in medication supply, financing and concerns regarding adverse events, as well as insufficient program reach and participation in some areas [14,15,17,19]. As a result, six non-consecutive rounds of MDA over an 11-year period were completed in the study area at the time of the surveys from 2004 to 2014 inclusive. The program has led to considerable reductions in LF prevalence in many areas of Myanmar [15,17], but areas of persisting transmission remain [15,17,19].

Two recent surveys assessed the cross-sectional prevalence of LF in the Mandalay Region, a remaining transmission hotspot, in 2014–2015 [6,19,20,21,22,23]. Their complete serological data have not yet been reported, nor has any investigation into the utility of each individual infection marker in post-MDA surveillance.

Results from both surveys contribute to the current study, which aims to first generate a representative, age-stratified prevalence estimate for the study area in 2015 by Mf, antigen and antibody (Wb123 and Bm14 Ab). Next, to investigate the predictive value and relationship between different infection markers. Finally, to use Bayesian networks to evaluate the most efficient and sensitive testing methods for post-MDA and post-validation LF surveillance.

## 2. Methods

### 2.1. Sample Collection

Samples for serological testing were collected in two surveys in Mandalay Region, Myanmar, in 2014 and 2015. Firstly, from a representative community household cluster survey of those one year and older residing in 24 villages across four townships (Amarapura, Patheingyi, Tada-U, Wundwin) from January to March 2015 [19,20]. Secondly, from the baseline of a cohort study of antigen-positive and antigen-negative young people aged 10–21 years in one of these townships (Amarapura) in October 2014 [21,22].

Fingerprick whole blood samples from participants in both surveys were tested immediately for circulating filarial antigen by Binax Now Filariasis ICT cards (Alere Inc., Waltham, MA, USA) read at 10 min. In survey 1, night blood Mf slides (60 μL total in 3 lines) were taken on ICT positives where possible [19]; no slides were possible in survey 2. Slides were stained with Giemsa and read by two blinded microscopists as described in Dickson et al. [19]. Fingerprick whole blood samples (60 μL) for later antigen and antibody testing from dried blood spots were taken in both surveys from all participants. Survey 2 was a matched sample of antigen-positive and -negative young people screened for the longitudinal study who were invited to a follow-up visit. Venous blood using EDTA anticoagulant was taken at the follow-up visit for plasma. Samples were handled and transported to Australia in July 2015, as previously described [21].

Both surveys collected information on demographics (age and gender), place of residence, and history of taking MDA in prior years. Information on whether MDA was taken at the most recent opportunity (2014 MDA for Survey 1 and 2013 MDA for Survey 2) was available for all participants. Information on ever-taking MDA was available for Survey 1 only.

### 2.2. Selection of Samples for Testing

Due to limited resources, antibody assays were done on an age-stratified sample (*n* = 376) of dried blood spots from survey 1. An age-stratified sample was selected to ensure sufficient sample size by age to compare antigen and antibody prevalence in younger age groups, as well as overall population prevalence of serology markers. The samples were classified by age category and randomly selected within these age strata using Excel random number generator, sorting by the resulting number, and selecting of the following. Set A comprises all children 14 years and under; 25% of those aged 15–29 y, 30–44 y, 45–59 y and ≥60 y by group. In addition, all remaining ICT positives in survey 1 (*n* = 33, i.e., those not randomly selected for SET A) were tested for Bm14 and Wb123 antibodies and are referred to as Set B. Set C comprises all samples from survey 2 that were tested for Bm14 and Wb123 antibody (*n* = 80 comprising 40 matched pairs (antigen positive and negative) based on gender and within one year of age).

### 2.3. Serological Testing

Og4C3 ELISA for antigen was performed on eluted fingerprick dried blood spots from both surveys to provide comparable results. The methodology of testing and positive/negative determination was as previously described [6]. Briefly, Og4C3 antigen was detected using the TropBio Og4C3 kits (Cellabs Pty. Ltd., Manly, Sydney, Australia) at 1:16 dilution. The kit supplies positive and negative controls with a cut-off for positivity recommended based on panels of negative control sera. A standard curve was constructed for each ELISA plate using the kit-supplied positive controls assigning an arbitrarily high value of 32,768 units with subsequent 4-fold dilutions. The Softmax Pro v5 software (Molecular Devices, Sunnyvale, CA, USA) was used to convert optical density readings of the test samples to units based on a four-parameter curve. A single cut-off point of >32 units (representing the sixth point of the standard curve from subsequent dilutions, corresponding to the kit recommended cut-off) was used to determine positive readings. The Og4C3 ELISA is less sensitive for dried blood spots (DBS) than serum or plasma, likely due to the recommended dilution for each sample type, which is approximately four-fold higher dilution for the DBS version [6].

Bm14 antibody assays were performed on eluted fingerprick blood samples in both surveys, using Cellabs kits (Cellabs Pty. Ltd., Manly, Sydney, Australia) protocols at 1:100 dilution with methods as previously described [23]. Briefly, a standard curve was constructed on each plate using a highly-positive sample from Papua New Guinea, which was defined by an arbitrarily high value of 1000 antibody units with subsequent 2-fold dilutions. A single cut-off point of >125 units was used to determine positive readings based on previous literature [5,9,24,25].

Wb123 antibody assays were performed using InBios kits (InBios International Inc., Seattle, WA, USA) as described in [26,27]. The kit provides positive and negative reference controls. In previous work, samples were classed as positive if their average optical density (OD) ratio was 9 or more times the negative control (based on the average ratio of the low positive 1:5000 serum control to blank), and results applied (for repeated control sera) to standard curves constructed using two-fold dilutions of a highly positive sample as described for Bm14. The tests were done at 1:50 dilution using eluted fingerprick samples from survey 1 (since there were no venous samples available) and EDTA plasma at same dilution for survey 2 samples.

### 2.4. Statistical Analysis

Ninety-five percent confidence intervals were estimated using the *proportion* command with the default *logit* option. Proportions were compared using the Chi-squared test in Stata 16 (Stata Corporation, College Station, TX, USA). Age and gender standardization were done using the *stdize* option in the *proportion* command using the 2014 census data by five-year age and gender groups for the Mandalay region (Myanmar Information Management Unit; http://themimu.info/ (accessed on 1 December 2021)).

Definition of outcomes:

(1) Antigen positive: Since the Og4C3 ELISA done with dried blood spots is less sensitive than ICT (see above), the definition of “antigen positive” was “positivity for ICT and/or Og4C3”.
(2) Antibody positive: samples positive for either or both Bm14 and Wb123 antibody was defined as “antibody positive”.
(3) Any infection marker positive: samples positive for at least one marker (antigen or either antibody) was defined as “any positive”.
(4) Missed positive: A “missed positive” was defined as a sample that had a negative result but was positive for one or more alternate markers.

### 2.5. Bayesian Network Analysis

#### 2.5.1. Bayesian Networks

**Figure 1:**
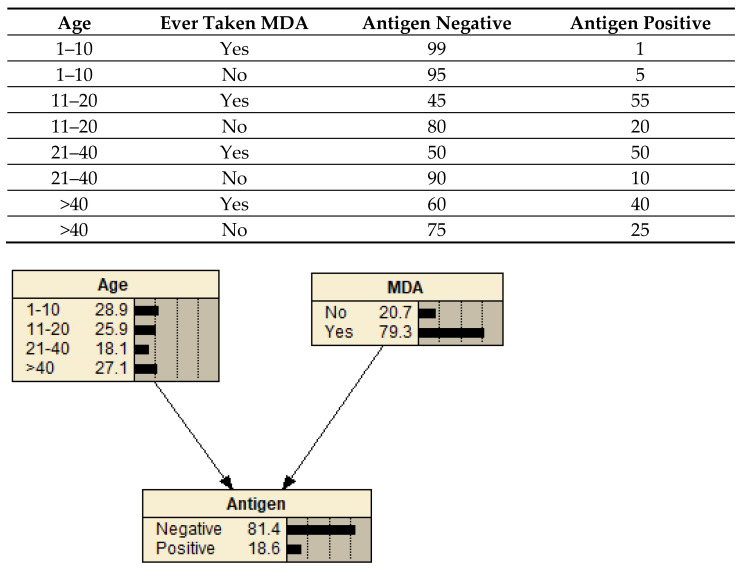
An example Bayesian network showing a sample conditional probability table (CPT) for the child node (Antigen) based on the possible state combinations of the parent nodes (Age and MDA). With the network in its default state, the probabilities shown in each node represent the evidence in the dataset, i.e., 27.1% of the population were aged >40 years, 79.3% took MDA, and 18.6% were antigen-positive.

Bayesian networks [28] are a graphical modeling method that represents the relationships between variables in terms of conditional probabilities. For variables (represented as nodes) that are dependent on another variable (the parent node), the conditional probability table (CPT) for each variable gives the probability of that node being in a given state based on the states of the parent nodes, for example, the probability that a sample is positive for antigen given a person’s age and whether they have ever taken MDA (Figure 1). Bayesian networks are increasingly being used in epidemiological studies because they are better suited than regression models for assessing complex systems and outcomes under different scenarios [29,30,31,32]. In this study, they were employed because they can efficiently compare the probability of a missed positive LF result with different diagnostic testing scenarios and assess the influence of various participant variables.

#### 2.5.2. Network Design

Bayesian networks were designed to assess the relationship between different infection markers and their utility in post-MDA surveillance.

Three separate networks were created in Netica (Norsys Software Corporation, Vancouver, BC, Canada): one for each infection marker as the respective outcome node. A naïve network structure (in which the outcome node was the only parent of every other node) was used to assess the effect of covariates (e.g., age) on outcomes. To facilitate scenario analysis, the structure was then modified with additional links: (a) between infection markers to evaluate their relationship, and (b) between ‘Ever taken MDA medication’ and ‘Took MDA medication last year’ to preserve causal logic (e.g., only participants who had ever taken medication could have taken it in the last year). The network structure was validated by researchers with expertise in both filariasis epidemiology and Bayesian networks.

#### 2.5.3. Network Testing

To evaluate the predictive validity of the Bayesian networks [33], each of the three models was tested using cross-validation. A random selection of 80% (*n* = 391) of the data was used to parameterize the CPTs of the Bayesian networks using the Entropy Maximisation algorithm. Model predictions for the remaining 20% of the records (*n* = 91) were then evaluated based on sensitivity, specificity and the True Skill Statistic [34], each of which used a classification cut-off of 50% (i.e., the option predicted as most probable was taken as the chosen outcome). A fourth metric, area under the curve (AUC) [35], was used to evaluate the models over a range of cut-offs. The four metrics were evaluated over 100 trials using a different random selection of records for each trial (with replacement between trials). Data from all three sample datasets were included in the analysis.

#### 2.5.4. Scenario Analysis

To examine the relationships between the three infection markers, each network was parameterized using all data from the combined dataset (*n* = 482). Networks were then used to estimate the probability of a missed positive case (i.e., negative result in a sample positive by other infection markers) during different diagnostic testing scenarios. Additional scenarios were conducted to assess the effect of participant factors and dataset on testing outcomes. Scenarios were undertaken by setting the node states of the relevant nodes and noting the probability of a positive test result on the outcome node (for example, setting the value of Wb123 Ab and Antigen to “negative”) to calculate the probability of a positive Bm14 Ab result.

## 3. Results

Table 1 describes the characteristics of each dataset and its participants. Dataset A and B originate from Survey 1. Survey 1 was a cross-sectional survey of individuals ≥1 year old across four townships. Dataset A included all individuals ≤14 years and an age-stratified sample (25%) of those ≥15 years. Dataset B incorporates all ICT-positive individuals not already included in Survey 1. Survey 2 was the baseline for a cohort study of age- and sex-matched antigen-positive and -negative young people aged 10–21 years in one of the townships included in Survey 1. The different sampling methods for each dataset resulted in variable median age, infection marker prevalence and MDA participation between datasets.

### 3.1. Infection Marker Positivity in the Overall Sample

**Figure 2:**
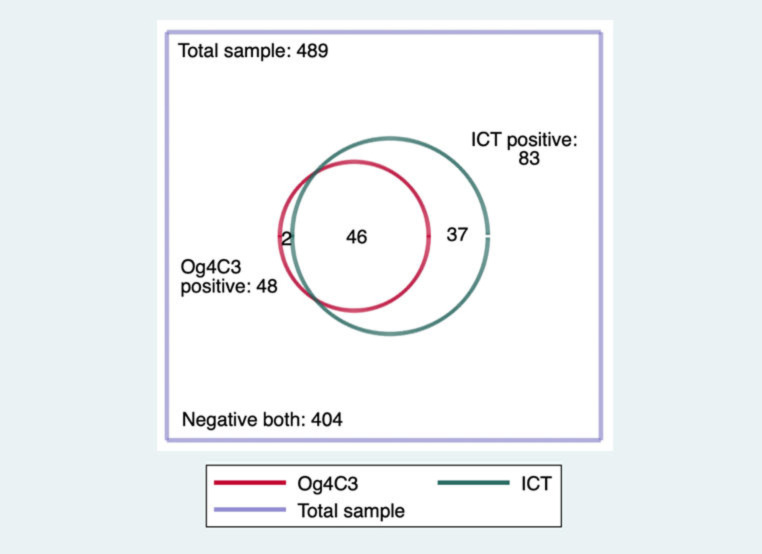
Number of samples positive by ICT and Og4C3 in the combined datasets, Myanmar, from 2014 to 2015.

Figure 2 shows a comparison of positivity by ICT and Og4C3 in the total sample of 489 tested from SETS A, B and C. It demonstrates the lower sensitivity of Og4C3 done using dried blood spots at the recommended dilution, which was seen to a similar extent in both surveys. Ag positive was therefore defined as either ICT and/or Og4C3 positive.

Overall, 85 of 489 samples were classed as antigen-positive (17.4%, 95% confidence interval (CI) from 14.3 to 21.0%). After standardization for age and gender, the estimated proportion positive for Ag in the whole sample was 18.1% (95% CI: from 14.6 to 22.2%).

**Figure 3:**
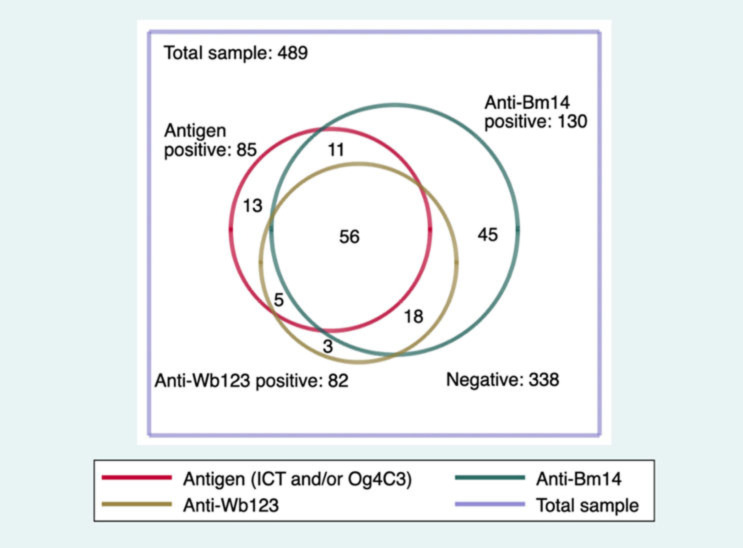
Numbers of samples positive for antigen and antibody in the combined datasets.

Figure 3 shows the antibody results. In the total sample (SETS A, B and C), the prevalence of antibodies to Bm14 (26.6%, 95% CI from 22.8 to 30.7%) was higher than for Wb123 (16.8%, 95% CI from 13.7 to 20.4%), which was of similar prevalence to antigen (17.4%, as noted above). For any antibody, 28.2% (95% CI from 24.4 to 32.4%) were positive, and 30.9% (95% CI from 26.9 to 35.1%) were positive for any marker of LF infection.

After adjustment for age and gender, the prevalence for Wb123, Bm14, any Ab and any positive were 17.4% (95% CI: from 14.0 to 21.5%), 27.9% (95% CI: from 23.7 to 32.5%), 29.6% (95% CI: from 25.3 to 34.2%) and 32.5% (95% CI: from 28.0 to 37.3%), respectively.

Combining SETS A and B, there were 47 Ag positive persons, of whom 42 had slides available; 17 of these were Mf positive, all for *Wuchereria bancrofti*. The Mf prevalence among Ag positives was 40.5% (95% CI from 25.6 to 56.7%). Seven of the thirteen antigen-positive, antibody-negative persons had slides taken, of which two (29%) were positive. No Mf were found in those under 15 years. Further details are given elsewhere [19].

### 3.2. Consumption of MDA Medication in the Overall Sample

Participants in all three datasets reported whether they had taken MDA at the last opportunity. The prevalence of LF markers of infection was much lower in those who had reported having taken MDA medication compared to those who had not (Figure S1). Datasets A and B also had information on whether participants had reported ever taking MDA, which demonstrated a similar association with LF markers.

### 3.3. Prevalence of Antigen and Antibody in the SET A Samples

The prevalence of Wb123 antibody was generally similar to antigen, while the prevalence for Bm14 or any antibody was higher. In the 376 persons sampled in SET A, overall crude antigen prevalence was 3.7% (95% CI from 2.1 to 6.2%). The corresponding crude prevalence for Wb123 Ab, Bm14 Ab, any Ab and any marker were 8.0% (from 5.6 to 11.2%), 18.9% (from 15.2 to 23.2%), 19.7% (from 16.0 to 24.02%) and 19.9% (from 16.2 to 24.3%), respectively.

After standardization for age and gender (necessary due to differing prevalence by age and gender, see below), the Ag prevalence in SET A was 4.4% (95% CI from 2.6 to 7.3%). The corresponding age and gender standardized prevalence for Wb123 Ab, Bm14 Ab, any Ab and any marker were, respectively, 8.7% (from 5.96 to 12.5%), 20.8% (from 16.0–26.6%), 21.9% (from 17.0 to 27.8%) and 22.5% (from 17.5 to 28.4%).

There were 230 females and 146 males in the SET A sample. The overall prevalence was significantly higher in males for antigen (6.2% versus 2.2%, *p* = 0.046), Bm14 Ab (24.7% vs. 15.2%, *p* = 0.023), any Ab (26.0% vs. 15.7%, *p* = 0.014) and any marker (26.7 vs. 15.7%, *p* = 0.009). A similar trend was seen for Wb123 Ab (10.3% vs. 6.5%, *p* = 0.191).

**Figure 4:**
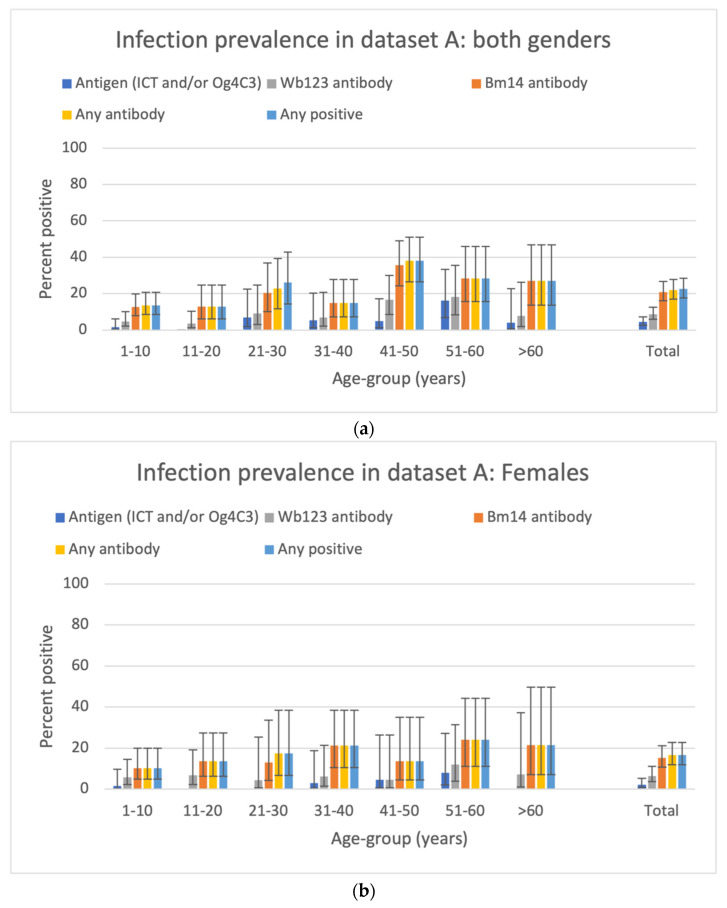

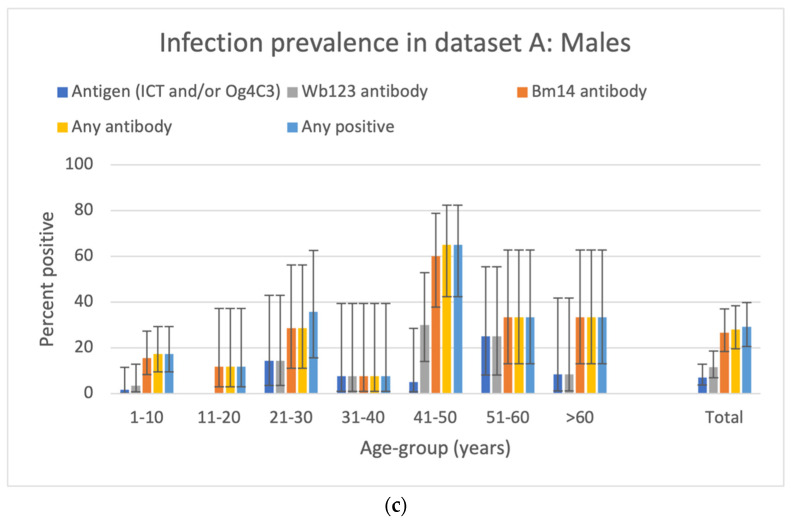
Age–specific prevalence for Ag and antibody markers by age group and overall for SET A samples. (**a**) Both genders; (**b**) females; (**c**) males. In (**a**), the total is standardized for age and gender. In (**b**,**c**), the estimates are standardized for age.

The age- and gender-specific prevalence of antigen in SET A are shown in Figure 4.

### 3.4. Bayesian Network Analysis for SETS A, B and C Combined

**Figure 5:**
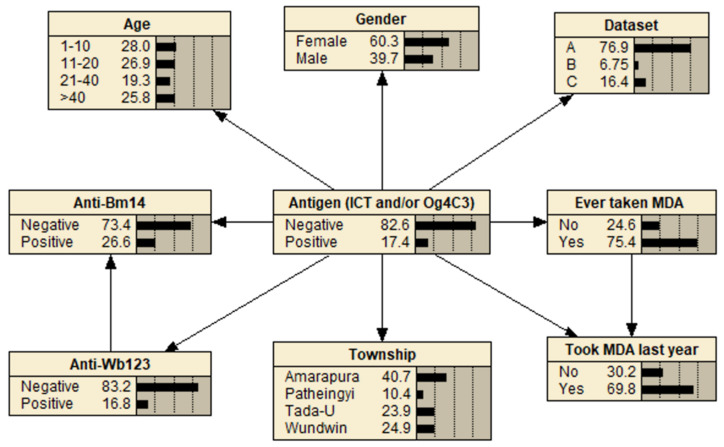
Bayesian network with antigen as the outcome node.

The three Bayesian networks used to evaluate the infection markers are shown in Figure 5 and Figure S2.

The average AUC and True Skill Statistic over 100 trials ranged from 0.80 to 0.97 and from 0.54 to 0.79, respectively (Figure S3). The performance of each network was considered sufficient to warrant their use in the scenario analysis. The Bayesian network for Bm14 Ab was less sensitive than the other two models, indicating that antigen and Wb123 were less able to predict Bm14 Ab.

**Figure 6:**
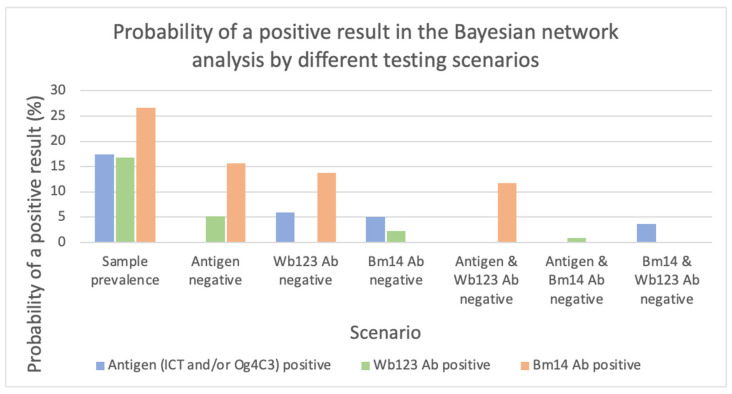
Probability of a positive result in the Bayesian network analysis if tested negative under different diagnostic testing scenarios.

Using the entire dataset, the probability of a positive test by different diagnostic testing scenarios is shown in Figure 6. Prior to defining any scenario, the probability of a positive result was 17.4%, 16.8% and 26.6% for antigen, Wb123 Ab and Bm14 Ab, respectively. If antigen was negative, there remained a sizeable probability of a positive result by Wb123 Ab (5.2%) and Bm14 Ab (15.6%). If either antibody was tested individually or together, and the results were negative, a considerable proportion was antigen-positive (5.9% for Wb123, 5.0% for Bm14, 3.7% for both). The testing combination of antigen plus Bm14 Ab detected the highest number of cases, with a probability of a missed positive as detected by Wb123 Ab of 0.88%.

Figure S4 displays the results of the different diagnostic testing scenarios by dataset and various participant factors (age, gender, township and history of MDA consumption).

By age, there was a higher probability of a positive result in older groups across all scenarios (Figure S4a). Of note, in the 1–10 age group, there was a greater discordance between Bm14 and both antigen and Wb123 Ab compared to older ages. Males demonstrated a greater probability of a positive result compared to females, but the relationship between markers was similar (Figure S4b).

Among townships, the likelihood of positive result was highest in Amarapura (the site of the cohort study) (Figure S4c). The concordance between Bm14 Ab and both antigen and Wb123 Ab was notably closer in Patheingyi and Wundwin compared to Amarapura and Tada-U.

Across all scenarios, the probability of a positive result was lower if MDA medication had ever been taken or within the last year (Figure S4d,e). Taking MDA medication was associated with a lower antigen and Wb123 Ab, relative to Bm14 Ab.

As expected, the representative sample (survey 1) had a lower likelihood of a positive result compared to the cohort baseline sample (survey 2), where half the individuals were antigen-positive (Figure S4f). All three datasets showed a moderate probability of antibody positivity if antigen-negative and that testing antigen plus Bm14 Ab resulted in the lowest probability of a missed positive result. Dataset C had a greater difference between antigen and Wb123 Ab, but each correlated more closely with Bm14 Ab.

## 4. Discussion

This study demonstrated a high seroprevalence of lymphatic filariasis in an area of Myanmar with ongoing transmission following several rounds of MDA. A Bayesian network framework was used to evaluate the relative utility of different infection markers for detecting signals of transmission in post-MDA surveillance. It found that antigen-based tests alone missed a considerable proportion of those with positive Wb123 and/or Bm14 Ab, including in the under-10 age group. Similarly, the antibody-based tests Bm14 and Wb123 alone, or in combination, also failed to detect some antigen-positive cases. The combination of antigen plus Bm14 Ab testing yielded the most sensitive marker of current or past infection. These findings suggest that post-MDA surveillance surveys would benefit from the addition of Bm14 Ab to more accurately detect potential signals of transmission and prevent the premature cessation of MDA.

Previous studies have reported on Bm14 antibodies in Myanmar for the SET C samples [23]. This paper is the first to report on Wb123 antibody prevalence and to compare antigen and antibody results for both Bm14 and Wb123 Ab in a population-representative sample (SET A). The high seroprevalence observed is similar to historic prevalence estimates in the region prior to the commencement of MDA [16]. The greater prevalence in males compared to females and with increasing age are consistent with those previously reported [25,36]. The marked antibody prevalence in males aged 41–50 years (60%) compared with a relatively lower prevalence in the 31–40 years group was unexpected and may reflect increased environmental exposure or sampling variation, although neither group was underrepresented.

A key finding of this study was that testing with antigen-based methods alone missed a sizable number of individuals with current or past infection. In post-MDA surveillance, the detection of any prior infection in children born since the commencement of MDA (11 years old in this context but 6–7 years for TAS surveys) is programmatically significant because it signals potential ongoing transmission. In the sample across all ages, the probability that antigen-negatives were positive for Wb123 or Bm14 Ab was 5.2% and 15.6%, respectively. Amongst those aged 10 years and younger, the relative difference between antigen and antibody tests was even greater.

The antibody tests for Bm14 and Wb123 alone, or in combination, also did not detect a considerable proportion of current infections in the Bayesian network. The finding of Mf in 29% of the antigen-positive, antibody-negative participants who had slides examined underscores the potential sensitivity limitations of these antibody assays. Bm14 Ab performed better than Wb123 Ab and detected most Wb123 positives (90%). The lower sensitivity of the Bm14 Ab Bayesian network signifies that antigen and Wb123 Ab were less able to predict Bm14 Ab and suggests that the Bm14 Ab may occur earlier during infection [8] or be more refractory to the effect of MDA. In contrast, Wb123 Ab tended to correspond with antigen tests across different diagnostic scenarios, including MDA consumption. This suggests that there are similarities between the expression of antigen and Wb123 during the filarial lifecycle and/or that they respond similarly to MDA.

The combination of antigen plus Bm14 Ab yielded the most sensitive marker of current or previous infection. This combination performed best both overall and across all subgroup scenarios. This suggests that Bm14 Ab testing should be considered for inclusion in TAS surveys to detect transmission more accurately and therefore prevent the premature cessation of MDA. Since Bm14 Ab is an ELISA-based assay that is not optimal for use in the field, a Bm14 rapid test will be required before it can be used in large-scale surveys. The available Brugia Rapid Test, which is used in non-*W. bancrofti* areas and detects antibodies to the similar antigen BmR1 [4], may provide a practical alternative. A comparison between Brugia Rapid and the Bm14 ELISA is needed before the rapid test can be recommended for use in TAS or similar surveys.

A notable finding was that MDA consumption was associated with a lower prevalence of all infection markers, with the greatest difference for antigen and Wb123 Ab. Antibody reductions after MDA have been observed previously [37] and probably relate either to a direct consequence of MDA’s filaricidal activity or to a lower probability of LF-exposure in areas where MDA uptake is higher. The greater discordance between Bm14 Ab compared to antigen and Wb123 Ab in Patheingyi and Wundwin townships in the current study likely indicates that transmission has reduced there to a greater degree than in Amarapura and Tada-U, where the markers remain more closely related.

These findings should be interpreted within the context of the study’s limitations. Firstly, resource constraints allowed for only a subset of participants from survey 1 to undergo serological testing, resulting in a conservative overall sample size (489). Secondly, the lack of clear timelines for longitudinal appearance and disappearance of each serological marker in individuals impedes clear conclusions regarding their specificity and sensitivity for detecting current infection. Thirdly, by defining a missed case as a negative result that was positive for one or more alternate markers, without the availability of a gold-standard test, the results do not account for the potential of false positives caused, for example, by testing cut-offs or parasite cross-reactivity. We also recognize that these samples were taken during 2014 and 2015 and may not reflect the current seroprevalence of infection markers. Nonetheless, the results from these robust Bayesian networks, across two separate surveys, demonstrate that either antigen or antibody testing alone may be insufficient for post-MDA surveillance.

## 5. Conclusions

This study has demonstrated that antigen or antibody testing in isolation is likely insufficient to detect transmission during post-MDA surveillance. The combination of antigen plus Bm14 Ab testing (or an equivalent antibody test) should be considered for inclusion in post-MDA surveys to increase the probability of detecting signals of transmission, particularly in low prevalence settings. Further studies are required to support this evidence and to evaluate comparable antibodies which are appropriate for large-scale field use.

## Figures and Tables

**Table 1:** Characteristics of the study and participants by dataset.

	Dataset		
	Survey 1		Survey 2
	A	B	C
Data Characteristics			
Study date	January–March 2015	January–March 2015	October 2014
Study type	Representative cross-sectional	Representative cross-sectional	Cohort
Study population	Community	Community	Community
Participant selection	Household members ≥ 1 y.o	Household members ≥ 1 y.o	10–21 y.o age- and sex-matched antigen positive and negative
Sample selection	≤ 14 y.o: all ≥15 y.o: age-stratified sample (25%)	All remaining ICT positives	All
**Participant Characteristics**			
Sample size	376	33	80
Median age (years) (interquartile range)	20.5 (9, 43)	46.5 (35.5, 57)	15 (12.5, 18.5)
Proportion female (%)	61	58	58
Township (n)	Amarapura: 94	Amarapura: 25	Amarapura: 80
	Patheingyi: 50	Patheingyi: 1	
	Tada-U: 112	Tada-U: 5	
	Wundwin: 120	Wundwin: 2	
Ever taken MDA medication (%)	81	64	– ^a^
Took MDA medication last year (%)	76	61	45
Antigen positive (ICT and/or Og4C3) (%)	3.7	100	47.5
Wb123 Ab positive (%)	8.0	69.7	36.3
Bm14 Ab positive (%)	18.9	69.7	45.0

^a^ Data not available.

## Data Availability

Data will be provided by authors upon request.

## Supplementary Materials

The following supporting information can be downloaded at: https://www.mdpi.com/article/10.3390/tropicalmed7070113/s1. Figure S1: Proportion of antigen and antibody positive by age group, shown by history of MDA taking last year; Figure S2: Bayesian networks with Bm14 Ab and Wb123 AB as the outcome nodes; Figure S3: Cross-validation testing of the three Bayesian Networks; Figure S4: Probability of a positive result in the Bayesian network analysis by different testing and subgroup scenarios.
