# Supplementary material for: Bayesian Network Analysis of Lymphatic Filariasis Serology from Myanmar Shows Benefit of Adding Antibody Testing to Post-MDA Surveillance
Source: Trop Med Infect Dis. 2022 Jun 21;7(7):113. doi: 10.3390/tropicalmed7070113 (PMC9323698; doi:10.3390/tropicalmed7070113)
Supplement: Supplementary file 1 [file tropicalmed-07-00113-s001.zip › tropicalmed-1728976-supplementary.pdf]

Tropical Medicine and Infectious Disease

MDPI

Bayesian Network Analysis of Lymphatic Filariasis Serology from Myanmar Shows Benefit of Adding Antibody Testing to Post-MDA Surveillance

Supplemental Figures

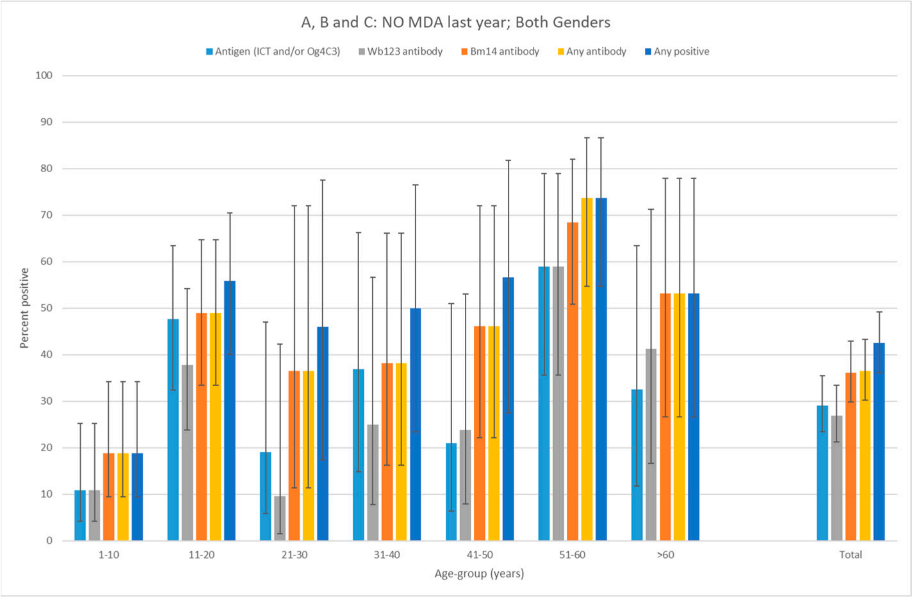

(**a**)

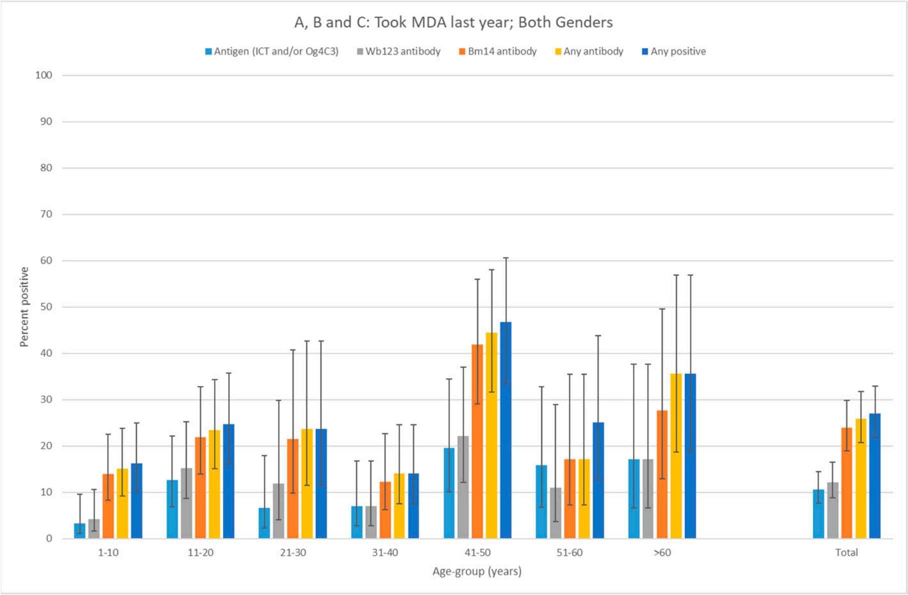

(**b**)

Figure S1. Proportion of antigen and antibody positive by age group, shown by history of MDA taking last year. (**a**): did not take MDA last year. (**b**): took MDA last year. Age specific prevalence are gender-standardized; totals are age- and gender-standardized. Includes data from all datasets (A–C).

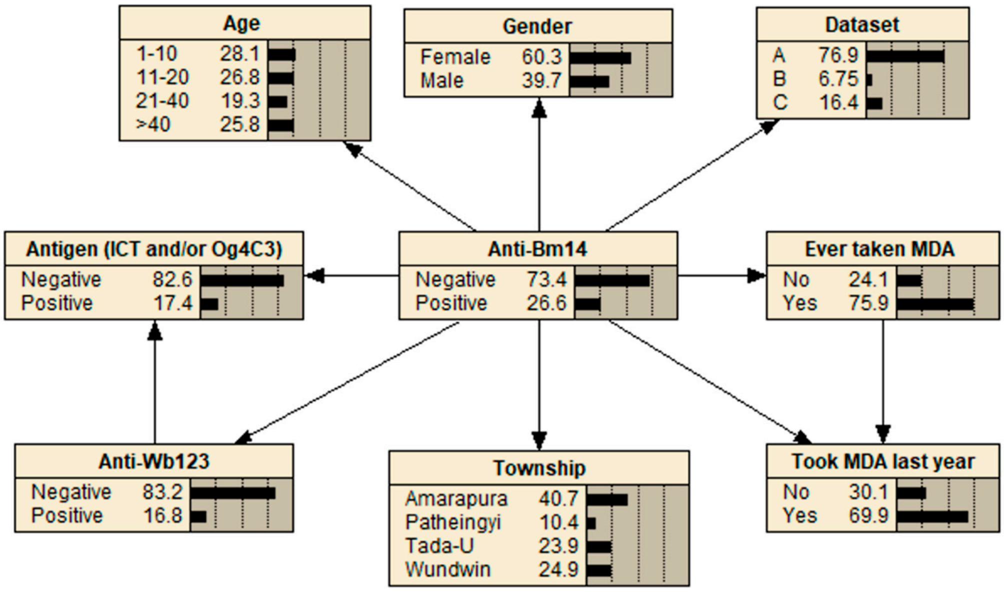

(**a**)

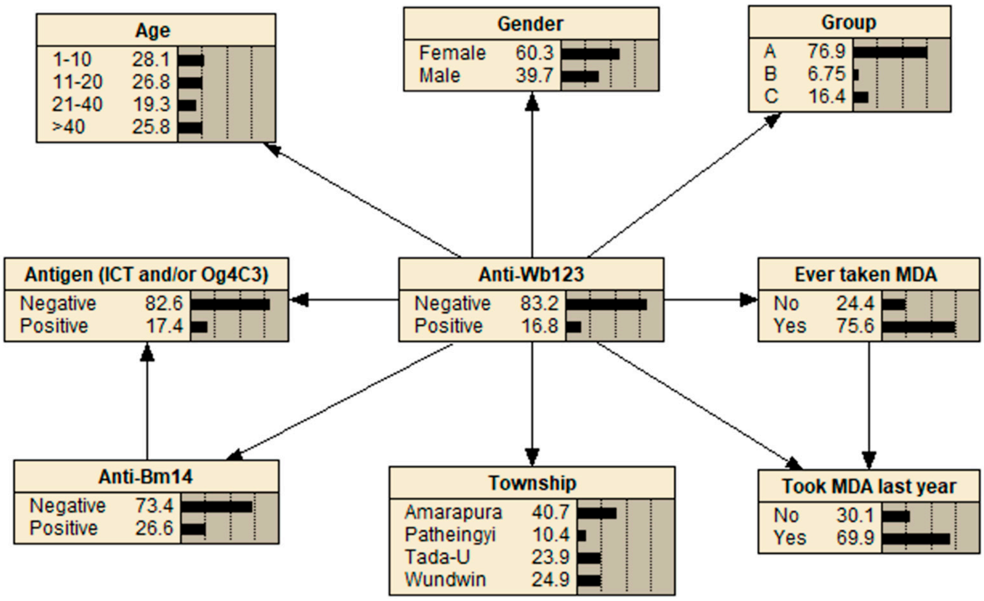

(**b**)

Figure S2. Bayesian networks with (**a**) Bm14 Ab and (**b**) Wb123 AB as the outcome node respectively.

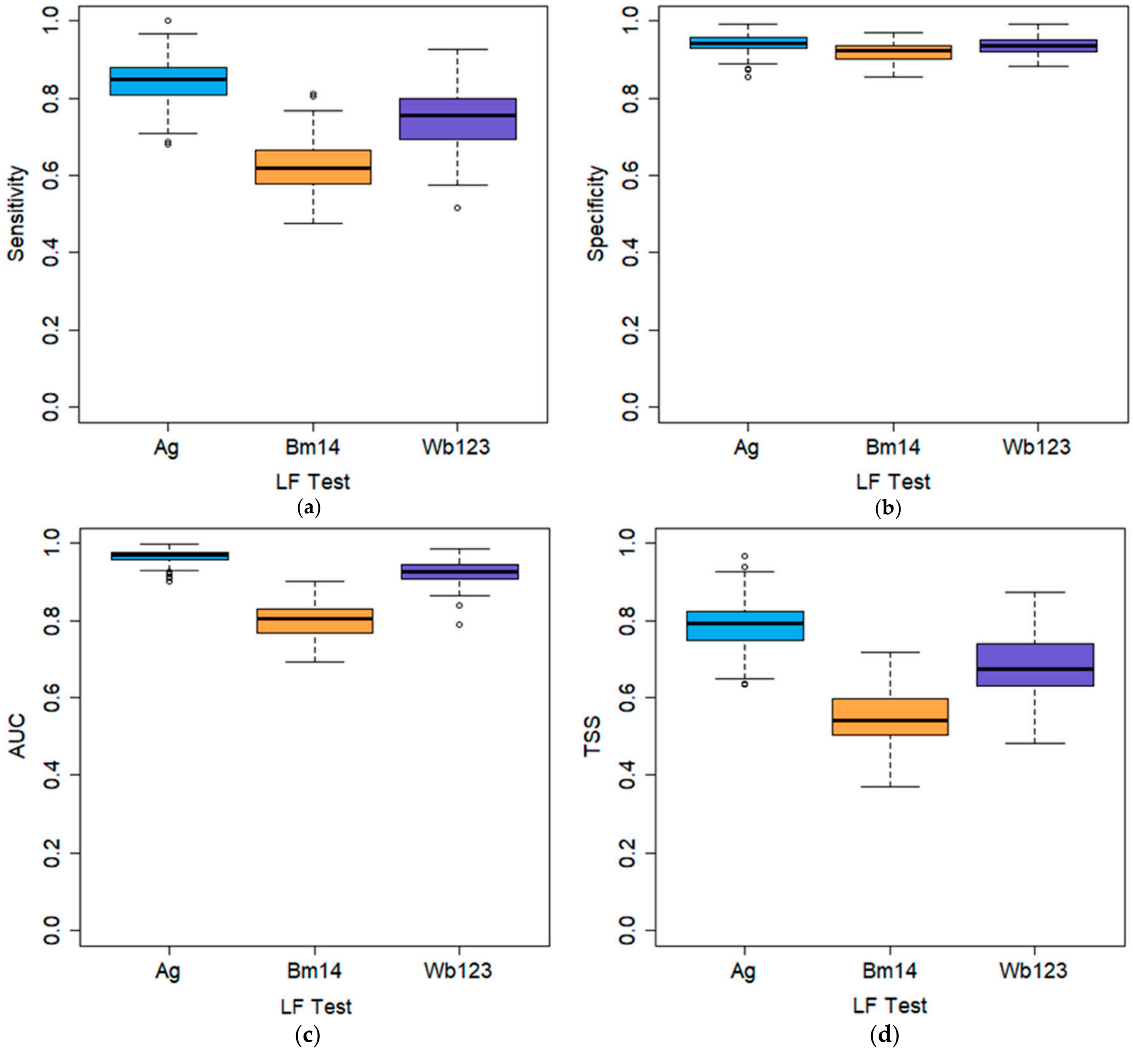

Figure S3. Cross-validation testing of the three Bayesian networks based on (**a**) sensitivity, (**b**) specificity, (**c**) Area Under the Receiver Operating Characteristic Curve (AUC) and (**d**) True Skill Statistic (TSS).

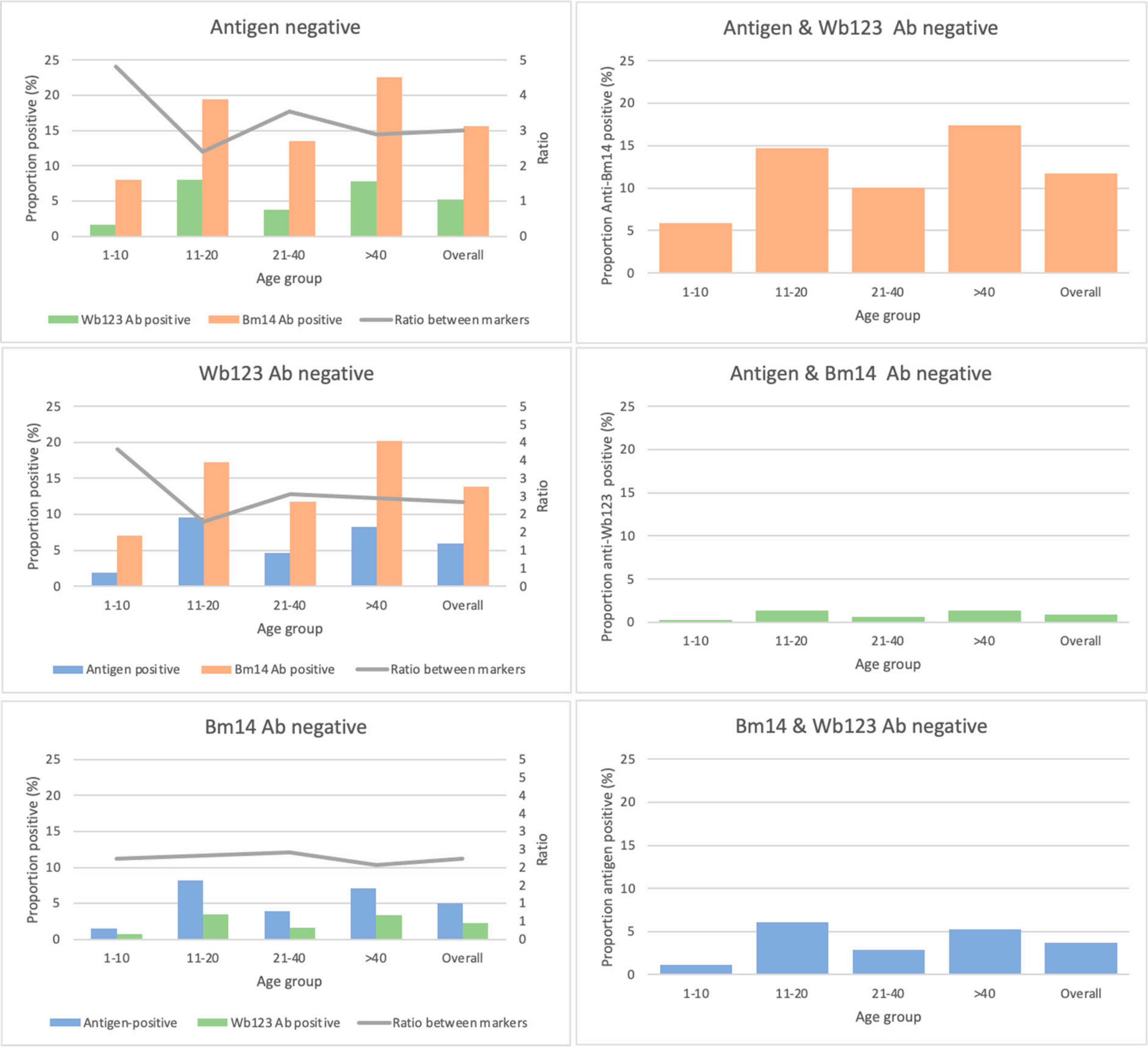
Antigen negative
Proportion positive (%)
Ratio
Age group
1-10
11-20
21-40
>40
Overall
Wb123 Ab positive
Bm14 Ab positive
Ratio between markers
Antigen & Wb123 Ab negative
Proportion Anti-Bm14 positive (%)
Wb123 Ab negative
Antigen positive
Antigen & Bm14 Ab negative
Proportion anti-Wb123 positive (%)
Bm14 Ab negative
Antigen-positive
Bm14 & Wb123 Ab negative
Proportion antigen positive (%)

(**a**)

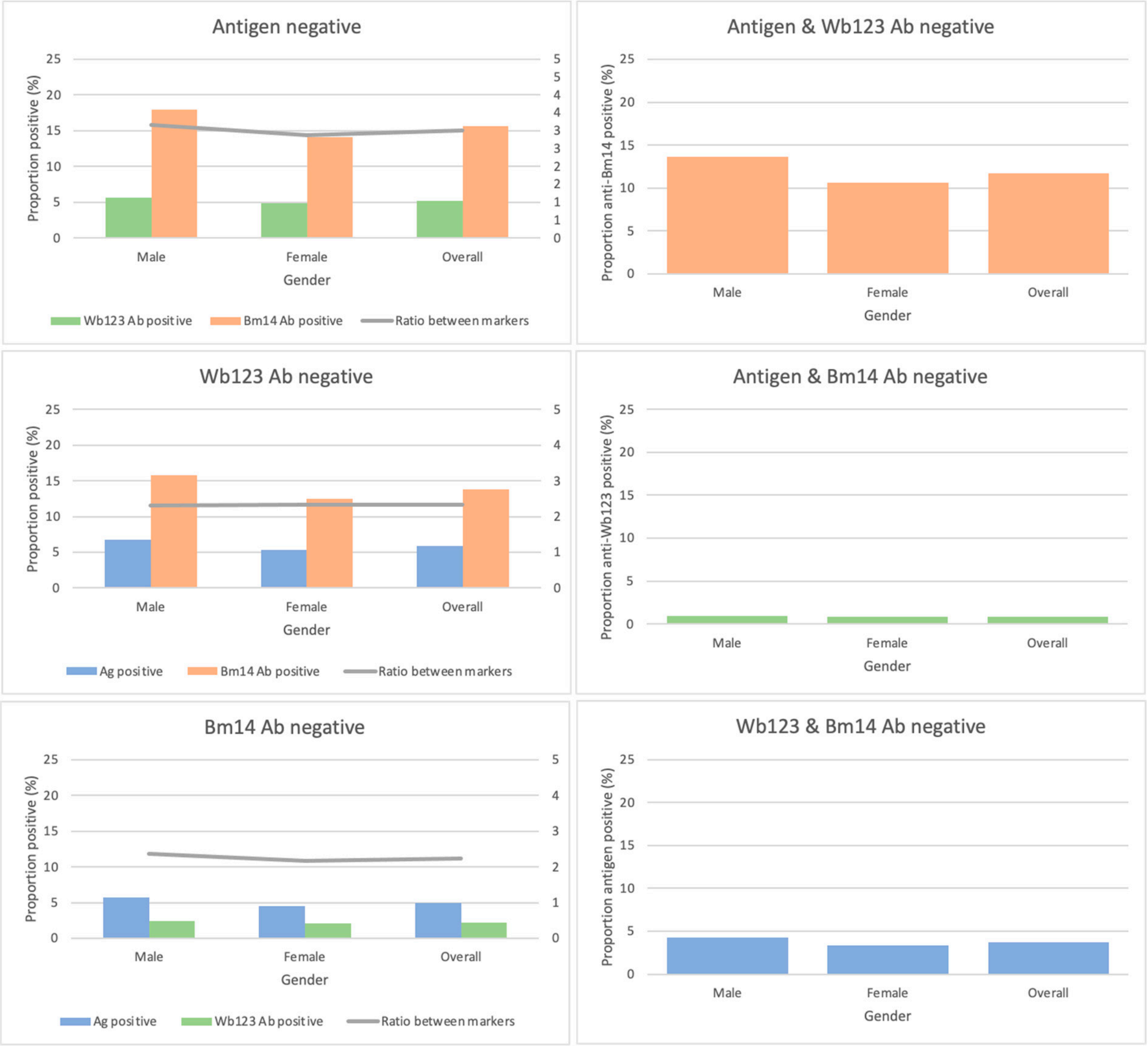
Antigen negative
Proportion positive (%)
25
20
15
10
5
0
5
5
4
4
3
3
2
2
1
1
0
Male
Female
Overall
Gender
Wb123 Ab positive
Bm14 Ab positive
Ratio between markers
Antigen & Wb123 Ab negative
Proportion anti-Bm14 positive (%)
25
20
15
10
5
0
Male
Female
Overall
Gender
Wb123 Ab negative
Proportion positive (%)
25
20
15
10
5
0
5
4
3
2
1
0
Male
Female
Overall
Gender
Ag positive
Bm14 Ab positive
Ratio between markers
Antigen & Bm14 Ab negative
Proportion anti-Wb123 positive (%)
25
20
15
10
5
0
Male
Female
Overall
Gender
Bm14 Ab negative
Proportion positive (%)
25
20
15
10
5
0
5
4
3
2
1
0
Male
Female
Overall
Gender
Ag positive
Wb123 Ab positive
Ratio between markers
Wb123 & Bm14 Ab negative
Proportion antigen positive (%)
25
20
15
10
5
0
Male
Female
Overall
Gender

(**b**)

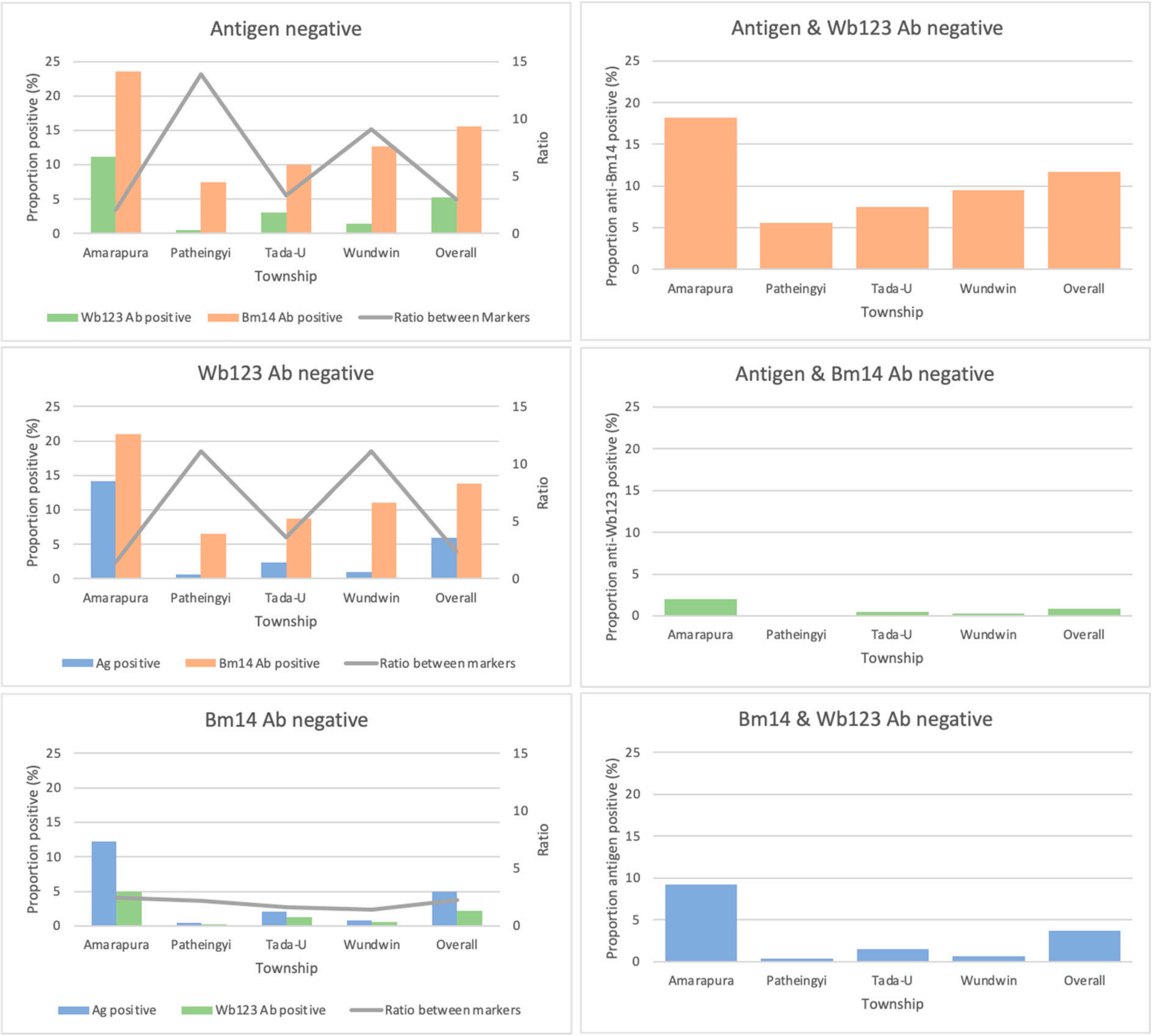
Antigen negative
Proportion positive (%)
Ratio
Amarapura
Patheingyi
Tada-U
Wundwin
Overall
Township
Wb123 Ab positive
Bm14 Ab positive
Ratio between Markers
Antigen & Wb123 Ab negative
Proportion anti-Bm14 positive (%)
Wb123 Ab negative
Ag positive
Ratio between markers
Antigen & Bm14 Ab negative
Proportion anti-Wb123 positive (%)
Bm14 Ab negative
Bm14 & Wb123 Ab negative
Proportion antigen positive (%)

(**c**)

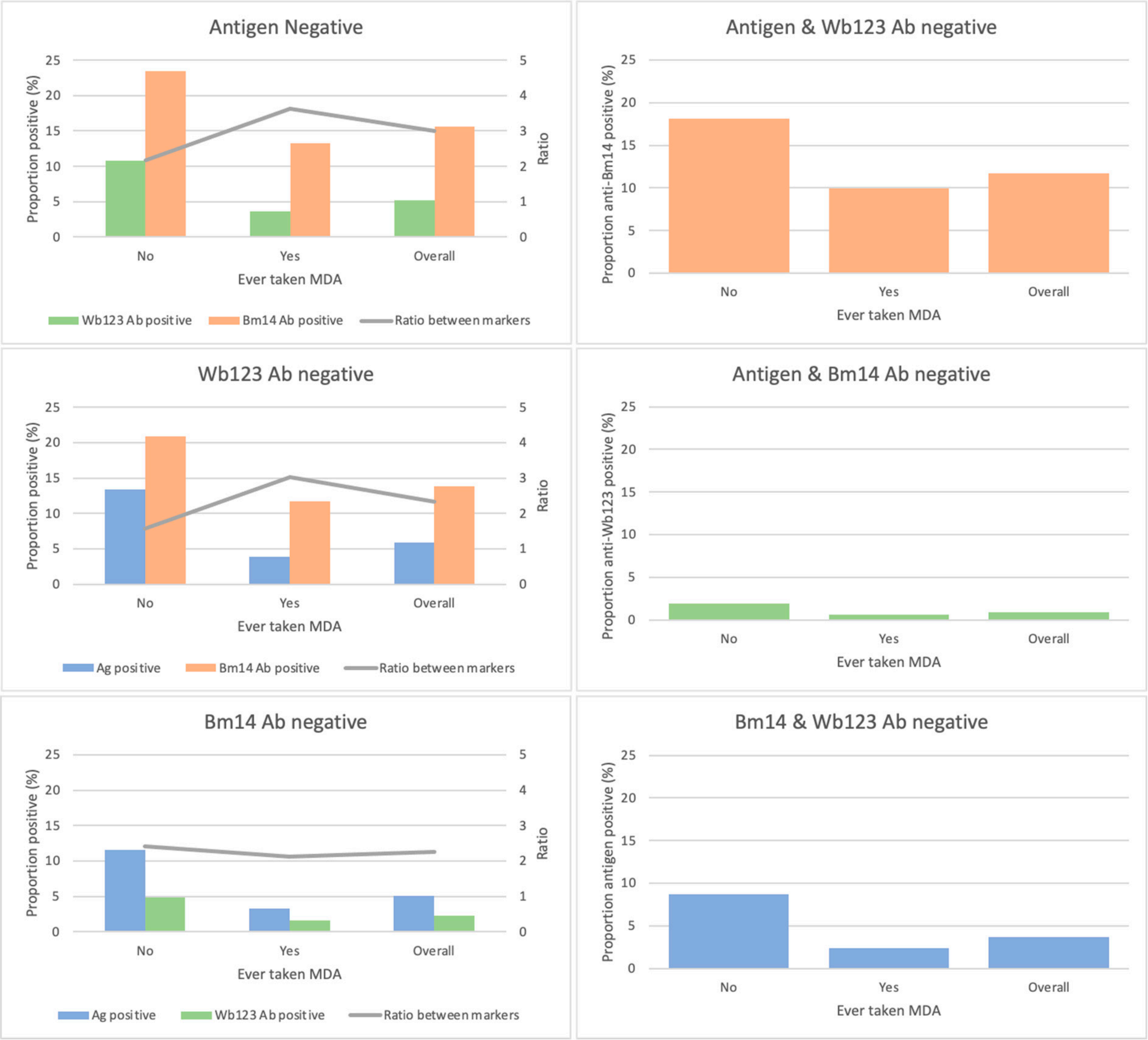
Antigen Negative
Proportion positive (%)
Ratio
No
Yes
Overall
Ever taken MDA
Wb123 Ab positive
Bm14 Ab positive
Ratio between markers
Antigen & Wb123 Ab negative
Proportion anti-Bm14 positive (%)
Wb123 Ab negative
Ag positive
Antigen & Bm14 Ab negative
Proportion anti-Wb123 positive (%)
Bm14 Ab negative
Bm14 & Wb123 Ab negative
Proportion antigen positive (%)

(**d**)

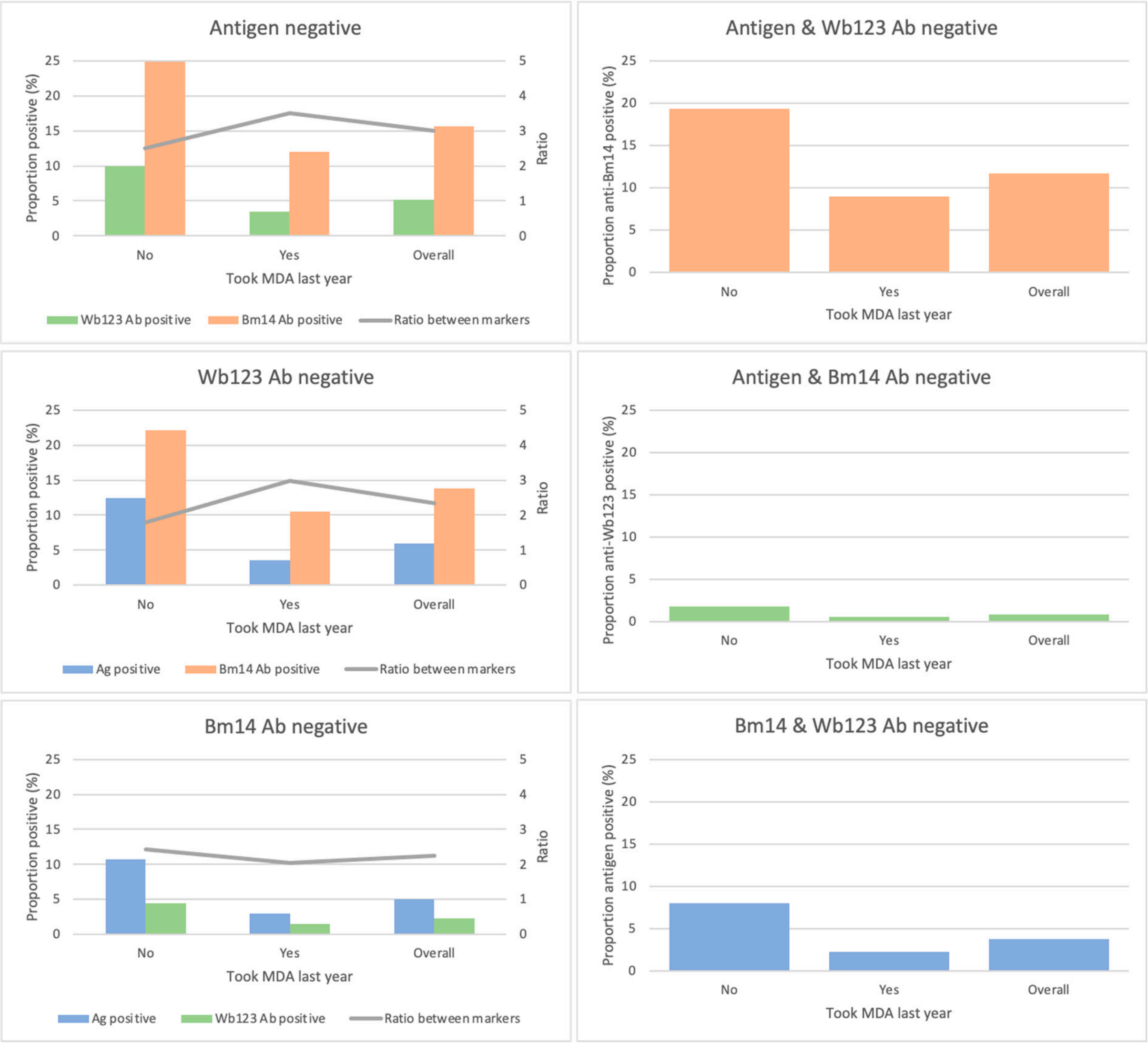
Antigen negative
Proportion positive (%)
Ratio
No
Yes
Overall
Took MDA last year
Wb123 Ab positive
Bm14 Ab positive
Ratio between markers
Antigen & Wb123 Ab negative
Proportion anti-Bm14 positive (%)
No
Yes
Overall
Took MDA last year
Wb123 Ab negative
Proportion positive (%)
Ratio
No
Yes
Overall
Took MDA last year
Ag positive
Bm14 Ab positive
Ratio between markers
Antigen & Bm14 Ab negative
Proportion anti-Wb123 positive (%)
No
Yes
Overall
Took MDA last year
Bm14 Ab negative
Proportion positive (%)
Ratio
No
Yes
Overall
Took MDA last year
Ag positive
Wb123 Ab positive
Ratio between markers
Bm14 & Wb123 Ab negative
Proportion antigen positive (%)
No
Yes
Overall
Took MDA last year

(e)

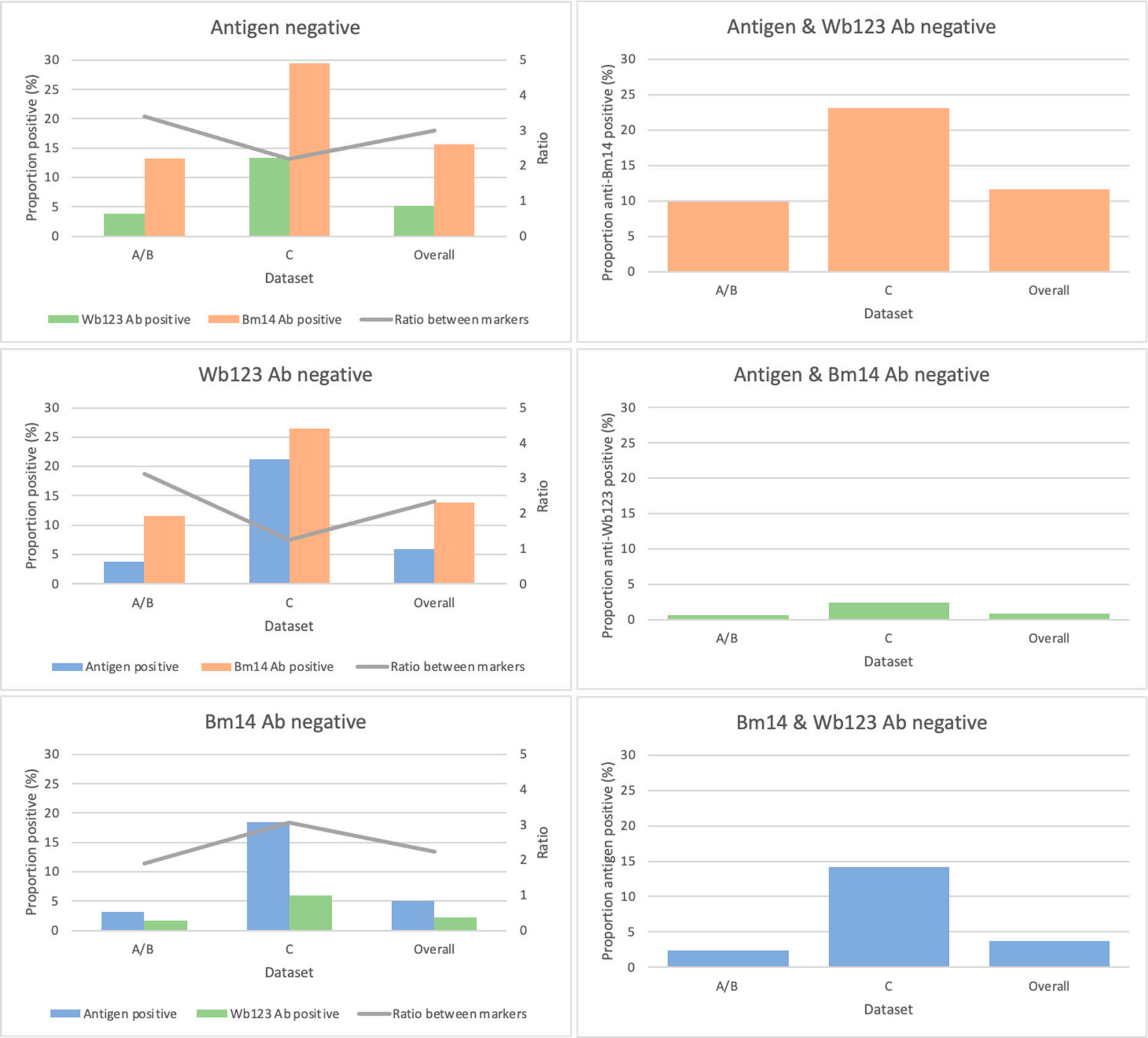

(**f**)

Figure S4. Probability of a positive result in the Bayesian network analysis by different testing scenarios and age group (**a**), gender (**b**), township (**c**), ever taken MDA medication (**d**), taken MDA medication last year (**e**) and dataset (**f**).
